# Safety Evaluation of the Aqueous Extract of the Mixture of *Persea americana* (Lauraceae), *Cymbopogon citratus* (Poaceae), Fruits of *Citrus medica* (Rutaceae), and Honey in Wistar Rat

**DOI:** 10.1155/jt/3145953

**Published:** 2025-09-15

**Authors:** Malane Nsangou Aicha El Ramadan, Bilanda Danielle Claude, Thamadeu Marie-Claire, Bidingha à Goufani Ronald, Tcheutchoua Yannick Carlos, Owona Pascal Emmanuel, Ngapout Fifen Rodrigue, Mbolang Nguegang Loick, Djientcheu Deugoue Franck Yvan, Dzeufiet Djomeni Paul Désiré, Kamtchouing Pierre

**Affiliations:** ^1^Department of Animal Biology and Physiology, Faculty of Science, University of Yaoundé I, Yaoundé, Cameroon; ^2^Department of Biological Sciences, Faculty of Sciences, University of Douala, Douala, Cameroon

**Keywords:** acute toxicity, *Citrus medica*, Cymbopogon *citratus*, honey, *Persea americana*, subchronic toxicity

## Abstract

As part of the valorization of traditional medicine drugs, toxicological studies were carried out on the mixture of the aqueous extract of *Persea americana, Cymbopogon citratus, Citrus medica*, and honey, a traditional mixture recognized for its antihypertensive activities. The experiments were conducted following modified OECD protocols 425 and 407 for acute and subchronic toxicity tests, respectively. For the acute toxicity, rats were divided into 3 groups of 6 rats (3 females and 3 males). They were given 2 single different doses of aqueous extract of the mixture and distilled water (2000 and 5000 mg/kg, 10 mL/kg). They were observed for the first 24 h and during 14 days. Clinical signs and nature of feces were evaluated. Concerning the subchronic toxicity, six groups of 10 animals each (5 males and 5 females) were used for each dose. The animals of group 1 received distilled water (10 mL/kg), and the animals of groups 2, 3, and 4 received the aqueous extract of the mixture (150, 300, and 600 mg/kg). The animals of groups 5 and 6 were normal satellite control and satellite extract 600 mg/kg. They received, respectively, distilled water (10 mL/kg) and the aqueous extract of the mixture (600 mg/kg). These last two groups were observed 14 days more than the other groups after complete cessation of all treatments. Clinical signs were evaluated and marked by normal fecal matter throughout the experimental period, with a nonsignificant weight variation. After the experiment, rats were sacrificed. For the subchronic toxicity evaluation, blood samples and some organs were taken. Relative organ was calculated, and hematological and biochemical parameters were also evaluated as well as histological sections of organs (pancreas, lungs, liver, kidneys, and spleen). Concerning the acute toxicity, some organs such as the pancreas, lungs, liver, kidneys, and brain were taken and weighed and relative weights were calculated. Administration of the aqueous extract of the mixture daily and for 28 days at the highest dose (600 mg/kg) led to significant increases in the weight of the kidneys (16.25%; *p* < 0.05) and lungs (58.72%; *p* < 0.001), and on the other hand, a significant drop in the relative weight of the spleen was observed (49.87%; *p* < 0.01). A significant increase in the level of leukocytes (*p* < 0.001) in animals of both sexes treated with the aqueous extract of the mixture at the highest dose was recorded. Treatment of animals with the extract at the highest dose resulted in a significant decrease in triglycerides (*p* < 0.05) in males. The microarchitecture of histological sections of the organs did not present any notable abnormality apart from a leukocyte shift at 600 mg/kg. The aqueous extract of this mixture is not toxic at the doses used traditionally (50, 100, and 150 mg/kg).

## 1. Introduction

Man has always used plants for multiple purposes: food, remedy, cosmetics, and edibles. Medicinal plants provide both an active ingredient and a multitude of compounds with complementary therapeutic effects [1]. Cameroon is among the African countries endowed with a rich floral diversity. According to the World Health Organization (WHO) [2], nearly 80% of the population in developing countries, including Cameroon, relies on herbal medicine for healthcare. This widespread use is attributed to the population's trust in traditional medicine, the accessibility of medicinal plants, and the recognition that these plants are valuable sources of bioactive compounds contributing to disease management. Dzeufiet et al. [3] scientifically demonstrated the antihypertensive effects of a mixture composed of fresh leaves of *Persea americana*, leaves and stems of *Cymbopogon citratus*, fruit of *Citrus medica*, and honey, in an alcohol- and sucrose-induced hypertension model.

Nevertheless, caution is required in the use of medicinal plants. Their excessive and uncontrolled consumption, without adequate safety evaluation, may expose individuals to serious adverse effects [4, 5]. Indeed, several studies highlight that medicinal plants are not devoid of risk [6]. For instance, cases of diarrhea and skin necrosis induced by certain medicinal plants have been documented [7]. Furthermore, the limited number of toxicological studies on medicinal plants increases concern regarding the toxic potential of plants for long-term use. It is therefore to respond to this problem that the evaluation of the toxicity of the aqueous extract of this mixture with antihypertensive power was carried out, which will be able to further legitimize the use of this extract and bring added value to it with regard to the development of an improved traditional drug.

## 2. Materials and Methods

### 2.1. Plant Material and Extraction

*Persea americana* leaves, leaves and stems of *Cymbopogon citratus*, and fruit of *Citrus medica* were harvested in the West Region, Cameroon, more precisely in the Noun division, in the Nkouh Seihn district. For honey, it was purchased from beekeepers in this same region on the same day. These constituents, with the exception of honey, have been identified in the National Herbarium of Cameroon. The identification of plant samples was made by comparisons to the existing sample numbers 18604/SRF/CAM, 18628/SRF/CAM, and 65106/HNC/CAM, respectively, for avocado leaves, lemongrass leaves and stems, and lemon tree leaves, respectively.

The quantities in grams of the different constituents of the mixture were, respectively, 70, 110, 300, and 500 for fresh *Persea americana* leaves, leaves and stems of *Cymbopogon citratus*, fruit of *Citrus medica*, and honey [3]. All plant materials, except honey, were rinsed with tap water and boiled in 2.5 L of distilled water for 40 min. The resulting decoction was decanted and filtered through Whatman No. 3 filter paper, and the filtrate was subsequently freeze-dried. In a previous study [3], the highest tested dose of this mixture was 150 mg/kg. For the present study, this dose was doubled and quadrupled to obtain 300 and 600 mg/kg, respectively. Thus, the three doses of 150, 300, and 600 mg/kg were used for subchronic toxicity testing.

### 2.2. Animal

Acute and subchronic toxicity studies were conducted on Wistar rats of both sexes, approximately 5 weeks old and weighing between 110 and 140 g at the beginning of the experiments. Males and females were housed in separate cages. All animals were maintained in the animal facility of the Faculty of Sciences, University of Yaoundé I (Cameroon), with free access to food and water. They were kept at room temperature under a natural 12/12 h light–dark cycle and grouped by three for the acute study and by five for the subchronic study. Animal handling complied with the European Community Directive (EEC 2010/63/EEC) on the protection of laboratory animals, and the protocol was approved by the Animal Ethics Committee of the Faculty of Sciences, University of Yaoundé I, as well as by the Cameroonian Bioethics Committee (registration no. FWA-IRB00001954).

### 2.3. Evaluation of the Acute Toxicity of the Aqueous Extract of the Mixture of *P. americana, C. citratus, C. medica*, and Honey

The evaluation of the acute toxicity of the aqueous extract of this mixture was done according to the modified OECD guideline 425 [8]. Eighteen nonwater fasted rats for 12 h (9 males and 9 females) were divided into 3 groups of 3 male and female rats each (2 tests groups 2000 and 5000 mg/kg of extract and 1 control group 10 mL/kg of distilled water). The blood glucose levels of each of them were recorded using an *accu check performa* glucometer. Each of the groups was treated by gavage in a single administration. The animals were observed individually at least once for 4 h following extract administration and then regularly for 24 h following administration and for 14 days following this treatment. Their appearance, the nature of their feces, their coat, their mortality, and their behavior (aggressiveness and sensitivity to touch and song) were regularly monitored. The animals were weighed twice per week, and the averages for each week were calculated. These animals were fasted at the end of each week to measure their blood glucose levels as described above. At the end of the 14-day experimental period, the animals were put on a nonwater fast for 12 h. They were anesthetized with ether and sacrificed. Organs such as the pancreas, liver, kidneys, lungs, and brain were removed. They were carefully examined and finally weighed, and their relative weights were calculated according to the formula [9]: RW = (OM/AM) ∗ 100 where RW is the relative weight of the organ; OM is the mass of the organ (g); and AM is the mass of the animal (g).

### 2.4. Evaluation of the Subchronic Toxicity of the Aqueous Extract of the Mixture of *P. americana, C. citratus, C. medica*, and Honey

This assessment was carried out following the modified OECD guideline 407 [10]. Six groups of 10 animals each (5 males and 5 females separate) were used. All animals received the substances daily by gavage. The work is done on the mixture of *Citrus medica*, *Cymbopogon citratus*, *Persea americana*, and honey. *Citrus medica*, *Cymbopogon citratus*, and honey are commonly used by the local population, for their breakfast as herbal tea. Since people tend to consume it in a large quantity, it is very likely that the dose of 150 mg/kg which is the therapeutic one is far under that amount. Therefore, that dose was multiplied by 2 and 4, respectively, for the toxicology study. The animals of group 1 received distilled water (10 mL/kg), and the animals of groups 2, 3, and 4 received the aqueous extract of the mixture of *P. americana, C. citratus, C. medica*, and honey (150, 300, and 600 mg/kg). The animals of groups 5 and 6 were the animals of the normal satellite control and satellite extract 600 mg/kg groups and received, respectively, distilled water (10 mL/kg) and the aqueous extract of the mixture (600 mg/kg). These last two groups were observed 14 days more than the other groups after complete cessation of all treatments. During this experimental period, the animals' body weights were recorded weekly and signs of toxicity were assessed.

### 2.5. Collection of Blood and Organs

At the end of the experiment, the animals underwent a 12-hour nonwater fasting period. They were then anesthetized with ether and sacrificed. Arteriovenous blood was collected into dry tubes and centrifuged at 3000 rpm for 15 min at 4°C, and the resulting serum was stored at −20°C for subsequent biochemical analyses. Additional arteriovenous blood samples were collected in EDTA tubes for hematological evaluation. Following blood collection, the rats were dissected, and organs including the pancreas, liver, kidneys, brain, lungs, and spleen were excised, rinsed with 0.9% NaCl solution, and weighed. The harvested organs were then fixed in 4% formalin solution for histological examination.

### 2.6. Biochemical Analyses

Glucose, total cholesterol, high-density lipoprotein, triglyceride, urea, uric acid, albumin, and total protein were determined by commercial diagnostic kits (LABKIT) by the calorimetric method [11]. However, the activities of alanine and aspartate aminotransferases (ALT and AST) and that of creatinine were evaluated by the kinetic method using the commercial diagnostic kits LABKIT and BIOLABO, respectively.

### 2.7. Hematological Assessment

The hematological parameters studied were white blood cells, red blood cells, platelets, hematocrit, hemoglobin, mean corpuscular volume, and mean corpuscular hemoglobin concentration. The evaluation of these parameters was carried out using a Mindray BC 3000 brand automatic analyzer.

### 2.8. Histological Evaluation

Concerning the histological analyses, they were carried out following the protocol of Smith and Bruton [12]. Slides were analyzed with ImageJ 1.3 software.

### 2.9. Statistical Analysis

The results were expressed as mean ± SEM. Statistical differences between control and treated groups were assessed by analysis of variance (ANOVA), followed by Tukey's multiple comparison test, using GraphPad Prism Version 8.01. Differences were considered statistically significant at *p* < 0.05.

## 3. Results

### 3.1. Acute Toxicity

#### 3.1.1. Evolution of Blood Glucose, Body Weight, Behavior, and Mortality of Animals

**Figure 1:**
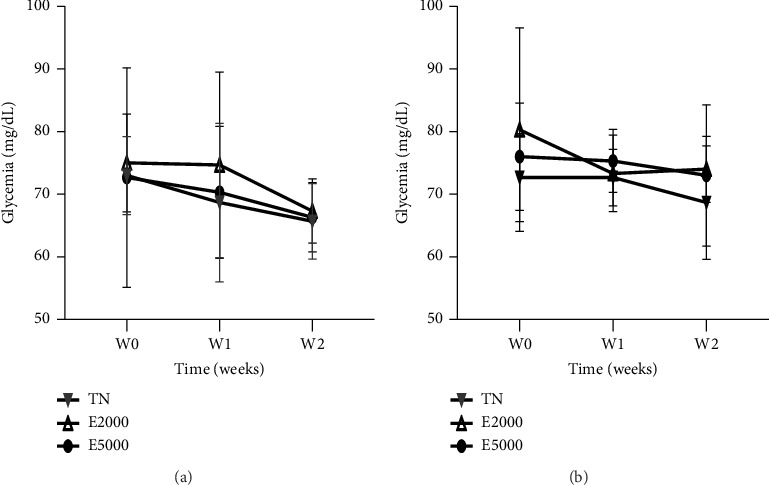
Evaluation of blood glucose levels of female (a) and male (b) animals during acute toxicity. Each point represents the mean ± SEM, *n* = 3. TN: normal rats receiving distilled water (10 mL/kg); E2000 and E5000: normal rats receiving aqueous extract of the mixture at doses of 2000 and 5000 mg/kg.

Figure 1(a) and 1(b) present the effects of the aqueous extract of the mixture on the blood glucose levels of the animals during the 14 days of the experiment. It appears from the figure that the administration of the aqueous extract of the mixture at single doses of 2000 and 5000 mg/kg compared to animals receiving distilled water (10 mL/kg) did not induce a significant variation in blood glucose levels which remained within normal ranges.

**Figure 2:**
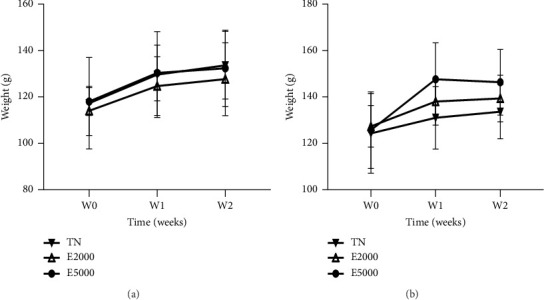
Evolution of the body weight of female (a) and male (b) animals during acute toxicity. Each point represents the mean ± SEM, *n* = 3. TN: normal rats receiving distilled water (10 mL/kg); E2000 and E5000: normal rats receiving aqueous extract of the mixture at doses of 2000 and 5000 mg/kg.

The effects of the aqueous extract of the mixture on body weight are compiled in Figures 2(a) and 2(b) in females and males, respectively. As for blood glucose levels, the administration of the aqueous extract of the mixture at single doses of 2000 and 5000 mg/kg did not reveal a significant variation in these animals compared to normal animals receiving water distilled (10 mL/kg).

Table 1 represents the effects of the aqueous extract of the mixture on mortality and some behavioral parameters of female and male animals. Single dose administration of the aqueous extract of the mixture at doses of 2000 and 5000 mg/kg did not induce mortality, even less change in behavior, nor sensitivity to noise or touch or even aggressiveness in animals receiving this extract at these doses compared to animals receiving distilled water (10 mg/kg).

#### 3.1.2. Evaluation of the Relative Weights of Some Organs

**Figure 3:**
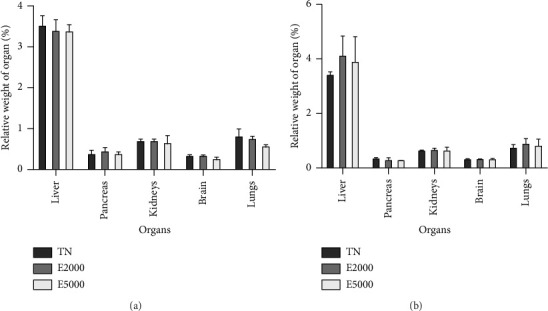
Evaluation of the relative weight of the organs of female (a) and male (b) animals during acute toxicity. Each bar represents the mean ± SEM, *n* = 3. TN: normal rats receiving distilled water (10 mL/kg); E2000 and E5000: normal rats receiving aqueous extract of the mixture at doses of 2000 and 5000 mg/kg.

Figures 3(a) and 3(b) present the effects of the aqueous extract of the mixture on the relative weight of some organs taken from female and male rats after the experimental period. From the following figure, the administration of the aqueous extract of the mixture at single doses of 2000 and 5000 mg/kg did not generate any significant variation in these organs either in females or in males.

### 3.2. Effects of the Aqueous Extract of the Mixture on Subchronic Toxicity

#### 3.2.1. Effects of the Aqueous Extract of the Mixture on the Evolution of Body Weight

**Figure 4:**
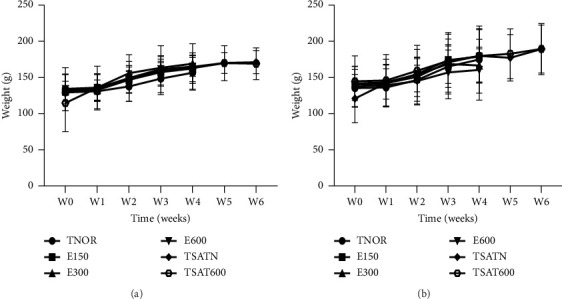
Effects of the aqueous extract of the mixture on the weight of female (a) and male (b) rats in subchronic toxicity. Each point represents the mean ± SEM, *n* = 5. TNOR: normal rats receiving distilled water (10 mL/kg); E150, E300, and E600: normal animals receiving the aqueous extract of the mixture at respective doses of 150, 300, and 600 mg/kg; TSATN: normal satellite observed 14 more days after stopping all treatments; TSAT600: satellite of the aqueous extract at a dose of 600 mg/kg observed for 14 days after stopping all treatments.

Figures 4(a) and 4(b) present the effects of the aqueous extract of the mixture on the body weight of female and male rats, respectively. Indeed, the administration of the aqueous extract of the mixture at doses of 2000 and 5000 mg/kg did not lead to a significant variation in weight in these animals, whether in females or in males receiving these doses of extract compared to animals of the same sex receiving distilled water (10 mg/kg).

#### 3.2.2. Effects of the Aqueous Extract of the Mixture on the Relative Weight of Some Organs

Table 2 summarizes the results of relative weights of some organs following subchronic toxicity in female and male rats, respectively. It appears from the table that in females, it was a significant reduction in the relative weight of the brains (11.05%; *p* < 0.05) in the TSAT600 group compared to the TSATN group. In males, administration of the aqueous extract of the mixture at a dose of 600 mg/kg led to significant increases in the weight of the kidneys (16.25%; *p* < 0.05) and lungs (58.72% *p* < 0.001) in the animals receiving this dose of extract, and on the other hand, a significant drop in the relative weight of the spleen (49.87%; *p* < 0.01) was observed in these same animals compared to the normal animals receiving distilled water (10 mL/kg). The aqueous extract of this mixture at a dose of 300 mg/kg in subchronic administration led to a significant reduction (38.47%; *p* < 0.05) in the weight of the spleen in animals receiving this dose of extract compared to animals receiving distilled water (10 mg/kg).

#### 3.2.3. Effects of the Aqueous Extract of the Mixture on Some Biochemical Parameters

Table 3 presents the effects of administration of the aqueous extract of the mixture on some biochemical parameters in female and male rats, respectively. It appears that, in females, the administration of the aqueous extract of the mixture did not induce any significant variation in the parameters studied in animals receiving the aqueous extract of the mixture compared to animals receiving distilled water (10 mL/kg). In male rats, as well as in females, no significant variation in the biochemical parameters studied was recorded in the animals receiving the aqueous extract of the mixture at different doses compared to those receiving distilled water except a significant reduction (27.35%; *p* < 0.05) in the concentration of triglycerides in animals receiving the aqueous extract of the mixture at a dose of 600 mg/kg compared to animals receiving distilled water (10 mL/kg).

#### 3.2.4. Effects of the Aqueous Extract of the Mixture on Some Blood Count Parameters

Table 4 summarizes the effects of the aqueous extract of the mixture on some blood count parameters in females and males, respectively. It appears that in females, the administration of the aqueous extract of the mixture at a dose of 600 mg/kg induced significant increases (31.77%, *p* < 0.001) of the leukocyte level compared to animals treated with distilled water.

In males, administration of the aqueous extract of the mixture at a dose of 600 mg/kg led to significant increases (78.39%, *p* < 0.001) of the leukocyte level in animals receiving this dose of extract compared to those receiving distilled water (10 mL/kg). As in females, administration of the aqueous extract of the mixture at a dose of 600 mg/kg also led to a significant reduction (14.55%, *p* < 0.05) in the mean corpuscular volume in animals receiving this dose of extract compared to those receiving distilled water (10 mL/kg). Furthermore, the administration of the aqueous extract of the mixture at a dose of 300 mg/kg also induced a significant increase (19.23%; *p* < 0.05) in the level of leukocytes in the animals receiving this dose extract compared to those receiving distilled water (10 mL/kg).

#### 3.2.5. Effects of the Aqueous Extract of the Mixture on the Microarchitecture of the Liver, Kidneys, Pancreas, Lungs, and Spleen in Male and Female Rats

**Figure 5:**
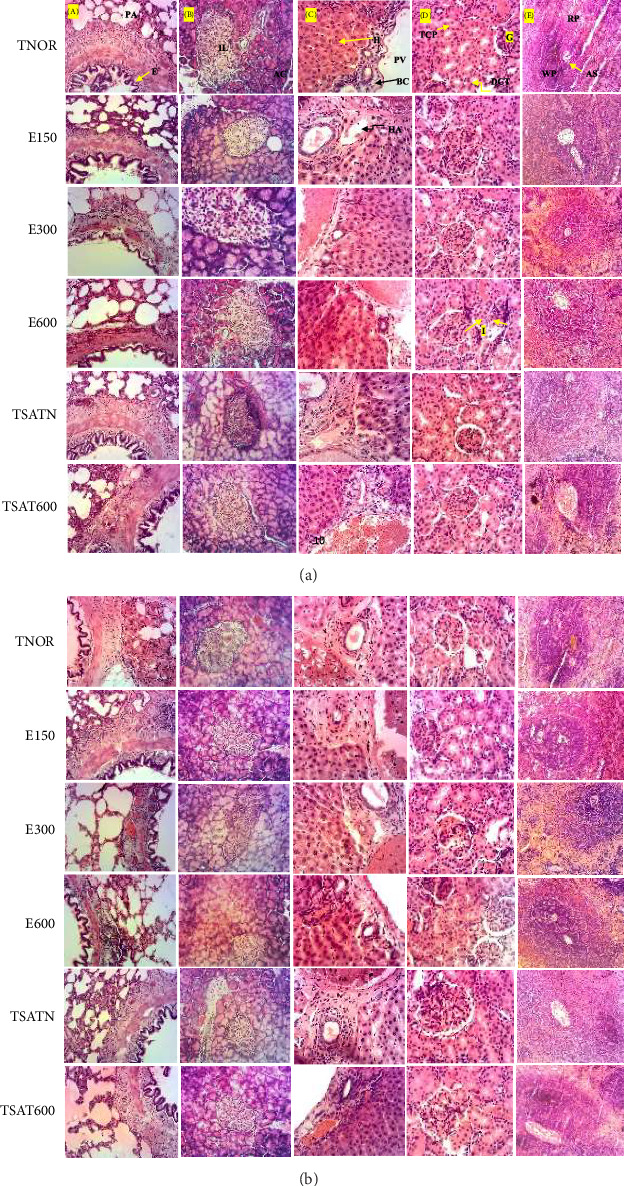
(a) Photomicrographs of sections of lung (A), pancreas (B), liver (C), kidney (D), and spleen (E) in male Wistar rats. Hematoxylin and eosin (100 ×); TNOR = normal control (distilled water 10 mL/kg); TSATN = normal satellite control (distilled water 10 mL/kg and observed 14 days after stopping all treatments); 150; 300; and 600 = aqueous extract of the mixture at respective doses of 150, 300, and 600 mg/kg; TSAT600 = satellite control receiving the plant extract at the highest dose (600 mg/kg) and observed 14 days after stopping all treatments; HA: branch of the hepatic artery; PA: pulmonary alveolus; AS: central splenic arteriole; B: bronchiole; AC: acinar cells; BC: bile canaliculus; E: epithelium of the pulmonary bronchiole; I: inflammation; IL: islets of Langerhans; H: hepatocyte; PB: white pulp; RP: red pulp; DCT: distal convoluted tubule; PCT: proximal convoluted tubule; VP: branch of the hepatic portal vein. (b) Photomicrographs of sections of lung (A), pancreas (B), liver (C), kidney (D), and spleen (E) in female Wistar rats.

Figures 5(a) and 5(b) represent histological sections of the lungs, liver, kidneys, and spleen in male and female rats. It appears that, in both males and females, apart from a slight leukocyte deployment, there was no major alteration in the pulmonary, pancreatic, hepatic, renal, and splenic tissues. For the spleen, in normal animals having received distilled water, the white pulp is made up of lymphocytic aggregates and shows a normal structural appearance. The red pulp which constitutes the bulk of the organ also presents a more or less normal structural appearance. There is no significant structural disorganization either in the red pulp or in the white pulp. Microscopic examination of pancreas sections from rats from the TNOR shows normal architecture of the pancreas with a normal appearance of the islets of Langerhans. The exocrine component forms a pancreas tightly packed by acinar cells and arranged in small lobules. The boundary between the exocrine and endocrine parts is quite clear. The pancreatic lobules are separated by intact intralobular and interlobular connective tissue septa, and islet cells are seen between the acinar cells. The islets appear slightly more colorful than the surrounding acinar cells. In the lungs, the architecture of the main branches of the pulmonary alveoli remains intact. For the kidneys, the glomeruli are well differentiated as are the urinary spaces. Concerning the liver of all these groups, the portal spaces can clearly be differentiated by the hepatic veins, the hepatic arteries, and the bile canaliculus.

## 4. Discussion

Any traditional drug, under normal conditions of use, is likely to produce side effects which may be allergic reactions, weight loss, variation in biochemical parameters, skin reactions, or damage to different organs such as the liver, kidneys, lungs, spleen, and central nervous system. In certain circumstances, the use of plants can also cause poisoning [13].

The acute toxicity study makes possible to observe the physiological parameters after administration of a large single dose of a substance which contains active metabolites. It manifests itself quickly and/or immediately after administration of the substance. The term acute toxicity is often closely linked to mortality. During our investigation, oral administration in a single dose (2000 or 5000 mg/kg) of aqueous extract of the mixture of *Persea americana, Cymbopogon citratus*, *Citrus medica*, and honey to rats of two sexes did not cause any death, no significant variation in blood glucose, and no atypical behavior (diarrhea, bristling of the coat nonreactivity to touch and noise, etc.) during the 4 h, 24 h, and 14 days after administration of the mixture at the doses of 2000 and 5000 mg/kg in animals of both sexes compared to those receiving distilled water. As a result, the lethal dose 50 (LD_50_) of this mixture is well beyond 5000 mg/kg and the mixture can be classified among nontoxic substances according to the Hodge and Stenner toxicity scale [14]. The present result does not correspond to those of Padilla-Camberos et al. [15] who showed that the ethanolic extract of the seed of *Persea americana* at a dose of 500 mg/kg already showed signs of acute toxicity. However, Ozolua et al. [16] showed that the LD_50_ of the aqueous extract of the seed of *Persea americana* was much higher than 1000 mg/kg. Similarly, the study carried out by Ayembilla et al. [17] showed that the LD_50_ of the ethanolic extract of *Cymbopogon citratus* leaves was greater than 5000 mg/kg in Sprague Dawley rats. Oyebadejo and Solomon [18] showed that a solution of 100% *Citrus* juice did not show signs of toxicity 24 h after its administration to Wistar rats. In addition, according to Islam et al. [19], honey contains 5-hydroxymethylfurfural, a compound not present naturally but which can be formed during heating or storage and which is cytotoxic, mutagenic, and carcinogenic. It appears that each constituent of the extract has varied and controversial toxicity limits in the literature; nevertheless, the mixture seems less toxic in light of the results we obtained. The evaluation of body weights as well as the organ relative weights of rats during acute toxicity showed no significant variation in these animals compared to those receiving distilled water. This supports the idea of the safety of this single-use mixture at the doses of 2000 and 5000 mg/kg.

The acute toxicity study is limited since we know that the bioaccumulation of a substance can cause serious harm even at very low doses. Therefore, the administration of multiples doses of a substance is necessary to evaluate its toxicological profile [20]. It emerges from the present investigation that, regarding the evaluation of the weights of female and male animals, they did not present any significant variation between the groups receiving the aqueous extract of the mixture at different doses (150, 300, and 600 mg/kg) and those receiving distilled water throughout the period of the experiment (28 days). Relative weight of organs is a more specific marker of toxicity than body weight [21]. This is why we have evaluated the relative weights of the organs which showed significant changes (kidneys +16.25%, lungs +58.72%, and spleen −49.87%) at 600 mg/kg in males. An increase in the relative weight of an organ is usually used as a predictor or indicator of pathological condition by toxicants [22]. So at this dose, the extract may not be safe for males.

As highlighted above, toxicity can disrupt biochemical parameters in animals [23, 24]. We then measured some parameters representing liver and kidney functions. Regarding liver parameters, ALT, AST, glucose, triglycerides, HDL-cholesterol, LDL-cholesterol, and total cholesterol are direct and indirect markers of liver function. ALAT and ASAT are intrahepatic enzymes capable of crossing the hepatocyte barrier and finding their way into the blood in the event of rupture of the hepatocyte membrane. The elevation of their activity is a good marker of damage to hepatocyte membranes [25, 26]. Their concentrations, as well as those of the other hepatic parameters studied, showed no significant variation in the rats receiving the aqueous extract of the mixture with three doses compared to rats receiving distilled water. These results corroborate those of Ozolua et al. [16] who did not show a significant variation in the level of transaminases during the study of the subacute toxicity of the aqueous extract of the seed of *Persea americana* in rats. These results were supported by the liver microarchitecture in these animals in which the hepatocytes appear regular and the portal spaces normal. However, triglyceride concentrations showed a significant drop in males from the E600 group compared to the TNOR group. This significant drop in triglycerides can be seen as a cardioprotective effect of this aqueous mixture on heart disease because the increase in triglycerides is a major factor in the development of atherosclerotic plaques [27]. This result is in disagreement with that of Ayembilla et al. [17] who showed a significant increase in the concentration of triglycerides in rats treated with the ethanolic extract of *Cymbopogon citratus* leaves.

Markers of kidney function were also assessed, including creatinine, urea, and uric acid. Any variation in these parameters would be a sign of renal damage [24]. No significant variation in these parameters was observed in animals of both sexes compared to rats receiving distilled water. This shows that the aqueous extract of this mixture cannot damage kidney function at the doses traditionally used. These results are in contradiction with those of Ekpenyong et al. [28] and Ayembilla et al. [17] which showed a significant increase in the concentration of creatinine during the oral administration of the infusion of the leaves of *Cymbopogon citratus* and a significant increase in the concentration of urea during oral administration of ethanolic extract of *Cymbopogon citratus* to rats, respectively. Overall, these results are encouraging and support the argument for the safety of this mixture at the higher therapeutic tested dose (150 mg/kg).

The main means of transport of drugs and xenobiotics is blood. During their transport, they come into contact with blood cells which then find themselves exposed if these substances are toxic. White blood cells are the body's defenders; their number can therefore vary depending on the toxicity or not of the drug [29]. In animals of both sexes from group E150 which is the therapeutic dose, no significant variation of leucocytes was observed. In animals of both sexes from group E600, we noted a significant increase in leukocyte levels and a significant decrease in mean corpuscular volume compared to animals from group TNOR. The increase in leukocyte levels is often associated with inflammation or stimulation of the immune system [29]. At this same dose, we observed in males the increase of relative weight of organ. The aqueous extract of this mixture at this dose could stimulate the immune system to defend body against inflammation. It would therefore not be prudent to use this dose for therapeutic reasons. This result is similar to that of Ayembilla et al. [17] who showed an increase in the number of leukocytes in animals receiving the ethanolic extract of *Cymbopogon citratus* leaves and is in disagreement with that of Nosiri et al. [30] who showed a decrease in the quantity leukocytes during their work. This can be explained by the solvent used for extraction that can lead to the different secondary metabolites.

## 5. Conclusion

It appears from the present work that up to the dose of 5000 mg/kg of the aqueous extract of the mixture, there was no sign of toxicity with the acute test. In the subacute study, no toxicity was also recorded at the dose of 150 mg/kg in oral and daily administration for 28 days. However, at the dose 600 mg/kg which is four times higher than this therapeutic dose, there were signs of inflammation and reaction of the body's natural barriers. The present results strongly suggest that mixture is safe at therapeutic doses but should be used with care.

## Figures and Tables

**Table 1:** Effects of the aqueous extract of the mixture on mortality and behavior of female animals.

Group	Sexes		Numb, Ani/group	Time	Behavior	Stool App	Aggressiveness	Mortality	Locom	Sen, Touch	Sen, Sound	Coat Condi
TN 10 mL/kg	F	M	3	1 h	Normal	Normal	No	No	Normal	Yes	Yes	Normal
	F	M	3	4 h	Normal	Normal	No	No	Normal	Yes	Yes	Normal
	F	M	3	24 h	Normal	Normal	No	No	Normal	Yes	Yes	Normal
	F	M	3	14 days	Normal	Normal	No	No	Normal	Yes	Yes	Normal

E 2000 mg/kg	F	M	3	1 h	Normal	Normal	No	No	Normal	Yes	Yes	Normal
	F	M	3	4 h	Normal	Normal	No	No	Normal	Yes	Yes	Normal
	F	M	3	24 h	Normal	Normal	No	No	Normal	Yes	Yes	Normal
	F	M	3	14 days	Normal	Normal	No	No	Normal	Yes	Yes	Normal

E 5000 mg/kg	F	M	3	1 h	Normal	Normal	No	No	Normal	Yes	Yes	Normal
	F	M	3	4 h	Normal	Normal	No	No	Normal	Yes	Yes	Normal
	F	M	3	24 h	Normal	Normal	No	No	Normal	Yes	Yes	Normal
	F	M	3	14 days	Normal	Normal	No	No	Normal	Yes	Yes	Normal

*Note:* TN: normal rats receiving distilled water (10 mL/kg); E 2000 and E 5000: normal rats receiving aqueous extract of the mixture at doses of 2000 and 5000 mg/kg. Numb. Ani/Group: number of animal/group, Sen: sensitivity to, App: appearance, Locom: locomotion, Condi: condition, F: female, and M: male.

**Table 2:** Effects of the aqueous extract of the mixture on the relative weight of some organs.

**Females**	**TNOR**	**E150**	**E300**	**E600**	**TSATN**	**TSAT600**

Liver	3.029 ± 0.199	2.932 ± 0.081	3.137 ± 0.294	3.131 ± 0.208	3.429 ± 0.249	3.248 ± 0.382
Pancreas	0.391 ± 0.065	0.386 ± 0.069	0.402 ± 0.097	0.433 ± 0.038	0.549 ± 0.079	0.549 ± 0.079
Kidneys	0.667 ± 0.028	0.622 ± 0.055	0.646 ± 0.074	0.610 ± 0.066	0.532 ± 0.009	0.557 ± 0.035
Spleen	0.508 ± 0.057	0.546 ± 0.014	0.619 ± 0.115	0.632 ± 0.063	0.376 ± 0.056	0.531 ± 0.088
Brain	1.026 ± 0.049	0.955 ± 0.059	1.026 ± 0.024	0.966 ± 0.033	1.055 ± 0.036	0.950 ± 0.047x^1^
Lungs	0.659 ± 0.015	0.707 ± 0.014	0.740 ± 0.030	0.619 ± 0.020	0.606 ± 0.054	0.579 ± 0.062

**Males**	**TNOR**	**E150**	**E300**	**E600**	**TSATN**	**TSAT600**

Liver	2.885 ± 0.176	2.901 ± 0.297	2.682 ± 0.232	3.054 ± 0.259	3.058 ± 0.593	3.284 ± 0.235
Pancreas	0.359 ± 0.026	0.299 ± 0.038	0.302 ± 0.041	0.298 ± 0.067	0.401 ± 0.037	0.409 ± 0.035
Kidneys	0.560 ± 0.022	0.632 ± 0.057	0.551 ± 0.070	0.651 ± 0.021 a^1^	0.643 ± 0.026	0.574 ± 0.034
Spleen	0.601 ± 0.099	0.469 ± 0.047	0.434 ± 0.089a^1^	0.401 ± 0.015a^2^	0.465 ± 0.088	0.430 ± 0.057
Brain	0.923 ± 0.086	0.782 ± 0.0768	0.928 ± 0.099	1.022 ± 0.159	0.992 ± 0.032	0.892 ± 0.072
Lungs	0.579 ± 0.036	0.734 ± 0.029	0.675 ± 0.072	0.919 ± 0.230a^3^	0.705 ± 0.038	0.717 ± 0.116

*Note:* Each value represents the mean ± SEM, *n* = 5. TNOR: normal rats receiving distilled water (10 mL/kg); E150, E300, and E600: animals receiving the aqueous extract of the mixture at respective doses of 150, 300, and 600 mg/kg; TSATN: normal satellite observed 14 more days after stopping all normal treatments; TSAT600: satellite of the aqueous extract of the mixture at a dose of 600 mg/kg and observed 14 more days after stopping all treatments; a^1^, a^2^, a^3^: *p* < 0.05; *p* < 0.01; *p* < 0.001: significant difference compared to normal animals that received distilled water of 10 mL/kg; x^1^: < 0.05: significant difference compared to the animals that received distilled water at the dose of 10 mL/kg for 28 days and observed for 14 days.

**Table 3:** Effects of the aqueous extract of the mixture on some biochemical parameters.

**Females**	**TNOR**	**E150**	**E300**	**E600**	**TSATN**	**TSAT600**

Creatinine (mg/L)	12.51 ± 0.14	13.13 ± 0.44	12.27 ± 0.19	12.49 ± 0.21	12.19 ± 0.54	12.80 ± 0.94
Urea (mg/dL)	46.10 ± 2.04	45.63 ± 2.87	44.53 ± 3.35	44.68 ± 0.77	44.8 ± 3.05	46.05 ± 3.22
Uric acid (mg/dL)	1.15 ± 0.06	1.05 ± 0.03	1.13 ± 0.03	1.28 ± 0.06	1.20 ± 0.02	1.32 ± 0.08
TC (mg/dL)	99.22 ± 3.60	93.66 ± 2.06	94.36 ± 3.95	103.50 ± 4.49	90.27 ± 2.33	88.28 ± 4.75
TG (mg/dL)	63.06 ± 2.55	55.73 ± 1.75	60.44 ± 4.26	56.82 ± 3.88	57.28 ± 4.19	62.55 ± 3.09
HDL (mg/dL)	49.13 ± 3.67	46.66 ± 2.67	47.94 ± 4.69	49.90 ± 2.46	47.31 ± 2.62	49.14 ± 3.14
LDL (mg/dL)	40.40 ± 4.97	33.85 ± 1.90	34.33 ± 2.81	39.54 ± 3.64	33.19 ± 1.95	29.21 ± 1.50
Albumin (g/L)	5.42 ± 0.12	5.064 ± 0.09	4.96 ± 0.14	4.73 ± 0.15	5.48 ± 0.31	5.18 ± 0.18
TP (mg/L)	0.77 ± 0.02	0.76 ± 0.03	0.75 ± 0.01	0.78 ± 0.03	0.73 ± 0.02	0.78 ± 0.02
ASAT (UI/L)	207.10 ± 11.28	204.90 ± 14.34	190.00 ± 11.00	212.30 ± 7.86	199.20 ± 7.94	226.60 ± 17.49
ALAT (UI/L)	48.07 ± 1.37	48.21 ± 5.09	44.01 ± 3.23	48.18 ± 3.95	55.58 ± 4.12	51.07 ± 6.09
Glucose (mg/dL)	73.99 ± 4.02	69.96 ± 2.34	77.45 ± 4.48	68.05 ± 1.59	80.89 ± 5.90	77.04 ± 4.56

**Males**	**TNOR**	**E150**	**E300**	**E600**	**TSATN**	**TSAT600**

Creatinine (mg/L)	12.29 ± 0.31	11.26 ± 0.21	11.58 ± 0.26	11.58 ± 0.26	12.00 ± 1.07	13.67 ± 0.21
Urea (mg/dL)	52.75 ± 2.40	57.10 ± 1.08	54.81 ± 3.17	64.91 ± 0.71	44.85 ± 4.07	49.46 ± 5.23
Uric acid (mg/dL)	1.08 ± 0.04	0.97 ± 0.06	0.97 ± 0.06	1.04 ± 0.05	1.14 ± 0.04	1.14 ± 0.04
TC (mg/dL)	95.63 ± 3.16	109.10 ± 5.67	108.30 ± 3.64	108.20 ± 2.15	103.70 ± 11.71	102.90 ± 5.59
TG (mg/dL)	64.15 ± 1.65	53.41 ± 4.48	60.85 ± 2.02	50.37 ± 1.52 a^1^	54.61 ± 0.95	52.77 ± 3.58
HDL (mg/dL)	35.45 ± 3.25	35.25 ± 1.22	32.74 ± 2.18	33.28 ± 2.79	33.25 ± 1.66	31.29 ± 1.04
LDL (mg/dL)	47.36 ± 4.01	54.38 ± 2.19	59.09 ± 2.28	60.70 ± 3.18	50.41 ± 5.38	52.69 ± 1.29
Albumin (g/L)	4.97 ± 0.20	4.90 ± 0.19	4.79 ± 0.10	4.66 ± 0.14	5.11 ± 0.12	4.68 ± 0.14
TP (mg/L)	0.71 ± 0.03	0.76 ± 0.01	0.75 ± 0.01	0.76 ± 0.01	0.69 ± 0.01	0.70 ± 0.01
ASAT (UI/L)	195.90 ± 6.04	183.50 ± 4.90	189.40 ± 6.86	177.90 ± 10.16	175.40 ± 6.60	179.90 ± 6.02
ALAT (UI/L)	49.00 ± 0.63	48.93 ± 0.37	54.95 ± 1.62	58.71 ± 0.67	55.55 ± 3.99	59.38 ± 1.36
Glucose (mg/dL)	77.67 ± 4.97	74.38 ± 2.74	67.01 ± 1.89	79.01 ± 4.24	85.36 ± 3.82	82.73 ± 6.09

*Note:* Each value represents the mean ± *SEM*, *n* = 5. TNOR: normal rats receiving distilled water (10 mL/kg);E150, E300, and E600: animals receiving the aqueous extract of the mixture at respective doses of 150, 300, and 600 mg/kg; TSATN: normal satellite observed 14 more days after stopping all normal treatments; TSAT600: satellite of the aqueous extract of the mixture at a dose of 600 mg/kg and observed 14 more days after stopping all treatments; a^2^, a^1^, *p* < 0.01; *p* < 0.05: significant difference compared to normal animals that received distilled water of 10 mL/kg.

**Table 4:** Effects of the aqueous extract of the mixture on some hematological parameters in female rats.

**Females**	**TNOR**	**E150**	**E300**	**E600**	**TSATN**	**TSAT600**

LEU (10^3^/μL)	7.90 ± 0.04	8.24 ± 0.13	8.23 ± 0.12	10.41 ± 0.23a^3^	7.68 ± 0.15	9.35 ± 0.80x^1^
PLQ (10^3^/μL)	424.00 ± 9.32	449.00 ± 8.14	414.00 ± 11.99	397.00 ± 27.54	441.00 ± 8.00	441.00 ± 5.99
RBC (10^6^/μL)	8.92 ± 0.06	8.95 ± 0.15	8.42 ± 0.25	8.77 ± 0.10	8.73 ± 0.24	8.89 ± 0.20
HEMA (%)	46.73 ± 1.99	47.95 ± 0.26	41.54 ± 0.39	42.84 ± 0.92	41.57 ± 0.29	43.62 ± 2.42
MGV (μL)	5.38 ± 0.47	5.57 ± 0.26	4.33 ± 1.06	4.84 ± 2.16	4.61 ± 2.13	4.06 ± 0.67
HB (g/dL)	15.03 ± 0.28	15.79 ± 0.09	14.48 ± 0.93	14.54 ± 0.28	14.56 ± 0.35	14.93 ± 0.63
TCMH (pg)	16.81 ± 1.03	17.64 ± 0.87	17.19 ± 1.38	16.67 ± 1.16	16.67 ± 2.22	16.79 ± 0.19
MCHC g/dL	33.76 ± 1.20	33.82 ± 0.08	31.53 ± 0.38	33.76 ± 1.16	33.10 ± 0.66	32.56 ± 0.68

**Males**	**TNOR**	**E150**	**E300**	**E600**	**TSATN**	**TSAT600**

LEU (10^3^/μL)	7.59 ± 0.07	7.66 ± 0.10	9.05 ± 0.33a^1^	13.54 ± 0.66a^3^	8.19 ± 0.20	8.03 ± 0.22
PLQ (10^3^/μL)	436.00 ± 6.75	453.00 ± 11.53	415.00 ± 2.70	458.00 ± 14.42	417.00 ± 0.71	467.00 ± 5.29x^2^
RBC (10^6^/μL)	9.19 ± 0.07	9.38 ± 0.14	8.65 ± 0.11a^1^	8.96 ± 0.16	9.10 ± 0.10	9.05 ± 0.13
HEMA (%)	40.90 ± 0.31	41.48 ± 0.53	34.79 ± 5.81	35.39 ± 0.51	39.04 ± 0.42	39.00 ± 0.54
MGV (μL)	4.50 ± 3.89	4.22 ± 1.93	4.21 ± 2.19	3.49 ± 0.80a^1^	4.90 ± 1.69	4.09 ± 3.17
HB (g/dL)	12.57 ± 0.30	13.91 ± 0.28	14.40 ± 1.17	14.56 ± 0.33	13.54 ± 0.32	14.93 ± 0.49
TCMH (pg)	13.67 ± 2.01	14.82 ± 1.63	16.64 ± 0.64	16.25 ± 2.52	14.87 ± 3.66	16.49 ± 1.26
MCHC (g/dL)	33.31 ± 0.85	32.15 ± 0.55	33.95 ± 0.48	31.14 ± 0.49	32.26 ± 0.14	32.65 ± 0.72

*Note:* Each value represents the mean ± SEM, *n* = 5. TNOR: normal rats receiving distilled water (10 mL/kg); E150, E300, and E600: animals receiving the aqueous extract of the mixture at respective doses of 150, 300, and 600 mg/kg; TSATN: normal satellite observed 14 more days after stopping all normal treatments; TSAT600: satellite of the aqueous extract of the mixture at a dose of 600 mg/kg and observed 14 more days after stopping all treatments; a^2^, a^3^: *p* < 0.01; *p* < 0.001: significant difference compared to normal animals that received distilled water of 10 mL/kg; x^1^, x^2^; *p*: < 0.05; *p* < 0.01: significant difference compared to the animals that received distilled water at the dose of 10 mL/kg for 28 days and observed for 14 days.

## Data Availability

The data are available from the corresponding author upon request.
